# Computer aided tool for diagnosis of ENT pathologies using digital signal processing of speech and stroboscopic images

**DOI:** 10.1186/2193-1801-1-64

**Published:** 2012-12-13

**Authors:** Amaia Méndez Zorrilla, Begoña García Zapirain, Agustín Pérez Izquierdo

**Affiliations:** 1 DeustoTech-LIFE Unit, DeustoTech Institute of Technology, University of Deusto 24, Bilbao, 48007 Spain; 2 Basurto Hospital, Bilbao, Spain

**Keywords:** Vocal pathologies, Otolaryngologist, Software application, Objective parameters, Diagnosis

## Abstract

The development of computer software and other technologies greatly facilitates the evaluation of pathological voice patients. This fact allows to reduce exploration time, improves the reproducibility of results and creates the possibility of test protocol standardization needed for the intercommunication between the different voice specialists. The proposed application encompasses the most important aspects which should be taken into account regarding dysphonic patients. It is a multidimensional scope which involves subjective questionnaires and perceptual, aerodynamic, acoustic and stroboscopic evaluations. In this system, the authors have designed and created simple tools for recording and automatic acoustic analysis for the acquisition and edition of stroboscopic images. The purpose is to work with all necessary tools running on a single application, without having to export and import data from other computer programs. Therefore, the objective is to synthetize the basic voice and the exploration of the vocal folds, simplifying it through the design of a program which helps us to analyze step-by-step each aspect of the vocal pathology. The evaluation of the tool has been performed by the otolaryngologists through periodical (medical) appointments on 25 patients for one year a year, and the results are promising either for the professionals as well as for the patients which receive a detailed report with the objective information concerning the features of their voice and vocal cords.

## Introduction

Considering the number of people suffering voice pathologies: between 3% and 9% in USA (Nerrière et al. [2009]), and 5% in Spain (SEORL, SEORL. http://www.seorl.net/. Accessed 26 [August 2012]), it is clear that these kinds of problems affect a very high percentage of the population. This is the reason why the authors believe that the study and development of techniques for the detection of vocal pathologies is important.

The advances in the area of diagnosis of otorhinolaryngology and speech therapy have been focusing in improving image acquisition devices, aimed at the observation of vocal folds and their functioning. Initially the ENT (Ear-Nose and Throat) specialist used a laryngeal mirror and performed the evaluation and diagnosis based on the information they could obtain with this device. Today we have other techniques for the acquisition and recording of images, which allow a posteriori evaluation, being Videolaryngostroboscopy (VLS) and Digital High-Speed videoendoscopy (HSV) the main ones.

It is important that the specialist knows the limitations the techniques used present, as VLS is only capable of capturing images at the speed of 25–30 frames/s (Lee et al. [2001]). These limitations affect mainly to the diagnosis of movement related pathologies and to the analysis of vocal fold vibration cycle, which in the case of VLS images it is only a human eye optical illusion.

However, HSV (Kiritani et al. [1986]; Kiritani et al. [1993]) captures images at the speed of 5000–8000 frames/s, thus providing a great amount of physiological and movement-related vocal fold information. Its general use is limited. There are very few hospitals which own the necessary hardware and the price is very high, unlike VLS. There is an intermediate solution called Videokimography (VKG) which provides high resolution images at a high speed with a more accessible price than VLS (Kim et al. [2003]).

Videolaryngostroboscopy is the most used technique for the diagnosis of some vocal pathologies (Braunschweig et al. [2008]) and the most common in the hospitals. Due to that reason, we have chosen it for the purpose of this study.

Against this background, it is necessary to provide objective parameters and tools for the practice of ENT specialists, not only in the area of vocal pathology diagnosis but also in the evaluation of the evolution of a rehabilitation process or a chirurgic intervention.

ENTs as well as speech therapists use more information than that obtained merely through image acquisition for their daily practice. Acoustic analysis is another one of their information sources, and although it does not serve as a diagnostic method by itself ([Hadjitodorov and Mitev 2002]), the pitch, jitter, shimmer and harmonics-to-noise ratio (HNR) parameters are accepted for the evaluation of voice quality and to measure the efficiency of the rehabilitation. Both techniques are used for diagnosis and treatment, as in the majority of cases people go to ENT and speech therapist appointments due to hoarseness.

The main aim of this work is to speed up the habitual practice of the specialists during the appointments through the automation of study methods, in response to the lack of applications which include audio and video processing functionalities together with the patient´s psychological aspect and result analysis.

The authors of this work have chosen VLS images (nevertheless assuming its limitations for the detection of some pathologies) because its use is widely spread in otorhinolaryngology (and the instrumentation is available in most hospitals), and focus in providing objective acoustic parameters plus VLS image information related mainly with organic dysphonia. This being the main aim, a number of secondary objectives are described as follows:

To design and develop a tool combining acoustic analysis and digital image processing which provides a report of final results according to the ELS (European Laryngological Society) guidelines.

To include different digital signal processing algorithms developed by the authors in previous works in the tool (Méndez et al. Mendez et al. [2009]; Mendez et al. [2012]), (García et al. [2009]).

The article is organized as follows: Section 2 describes materials, methods and functionalities of the designed and evaluated software. Section 3 shows the experimental results of the otolaryngologists in their daily practice using the application, and finally the authors offer concluding remarks in Section 4.

## Materials and methods

In this section the technical and human resources used during the development of the application are described, together with the methodology followed in its design to provide the professional with a useful tool for the daily practice.

### Technical resources

The proposed software is developed using a combination of Java (for the user interface) and Octave (to develop the signal processing algorithms). Among the elements more widely used for the implementation of this software are databases developed in XML (eXtensible Markup Language) and image processing libraries such as JMF (Java Media Framework). The necessary hardware is detailed as follows:

1. PC with integrated or external video capture device.
2. Stroboscope
3. Endoscopic Camera
4. Microphone for PC

### Human resources

In this study, 25 patients have participated during a year and all the obtained data has been registered. 18 of the 25 patients included in the study were women (72%), while 7 were men (28%), with an average age of 42, being the youngest 19 and the oldest 58 (standard deviation 11,43). All the patients whose sessions (voices and images) are recorded in the Hospital have signed the ethical consent to be included in this study.

### Methodology

One of the aims of the developed tool is to provide the professional (in this case medical) with openness regarding where the data resides and how does the system work with it. In order to fulfill that purpose the interface has been designed as simple as possible, with a pleasant user-friendly visual aspect and easy to use for not technical potential users.

The design of the application (made in collaboration with otolaryngologists) encompasses all the tests to be done during an appointment for voice exploration, which are organized following a comprehensive sequential structure.

This design follows the structure proposed by the protocols (Teatinos) as well as other internationally accepted standards such as VHI (Rodríguez et al. Rodríguez-Parra et al. [2009]), Dejonkere (ELS), all assumed by the SEORL (Spanish Society of Otorhinolaryngology and Cervico-facial Pathology) (Nuñez Batalla, Núñez [2007]) in its Basic Protocol for the functional evaluation of vocal pathology. The Basic Protocol proposes five basic steps: subjective analysis, perceptual analysis, acoustic analysis, aerodynamic analysis and stroboscopy.

The application, as it has been previously mentioned, was developed with the focus on the study of organic dysphonia. Numerous reasons exist for the appearance of dysphonia, and when treatment is applied on time it can help prevent worse ailments. The organic disphonyas are listed hereafter:

- Respiratory diseases: chronic laryngitis and bronchitis, asthma, adenoids, sinusitis, tonsillitis.
- Laryngeal malformations.
- Surgeries: tracheostomy, excisions of nodules or polyps, intubations.
- Laryngeal trauma. Produced by yelling often.
- Breathing and vocal misuse. By use of a faulty breathing technique, or the continued use of a tone that does not correspond to their organic characteristics.
- Trauma: frigths, abandonment, accidents.
- Characteristics of behavior: hyperactivity, shyness, inhibition.
- Family and social environment: family screaming, sport competitions, recreation, environments with excessive noise.
- Changes in hearing.

#### Personal data

In order to store a full record of the patient, as in any other query, a patient anamnesis must be performed containing the following information: name and surname, age, sex, personal and family medical history, toxic habits. The case history data and the examinations are stored and classified according to the date of the medical visit.

#### Subjective analysis

The subjective analysis section includes the Voice Handicap Index (VHI) questionnaire −30 (Weigelt et al. [2004]). It is a valid instrument for the assessment of the harm associated with dysphonia as perceived by the patient. The questionnaire is presented in short and full versions. In order to perform the numerical calculation of subjective analysis, this was divided into two general parameters: Impact and Vocal Quality.

The VHI contains 30 items organized into three groups of 10, called *physical subscale*, *emotional subscale* and *functional subscale*. It has been subsequently proved that these subscales are not separate measurements of speech impairment and that they have no validity as such.

Hsiung (Hsiung et al. [2002]) studied the correlation between measures of the voice laboratory and the results of VIH in patients with dysphonia; a large discrepancy between the two assessments was evidenced, which served as the basis to infer that a patient's feelings about a voice problem cannot be evaluated through objective measures.

The short form questionnaire is composed of two questions: “How do you think is the quality of your voice?”, and “how does it affect your social life or work?”.

#### Voice analysis

Voice Analysis is divided into the recording of the voice of the patient for the purpose of processing, and the calculation of the parameters of voice quality: pitch, jitter, shimmer and signal to noise ratio (García et al. [2009]). Furthermore, a perceptual analysis is performed.

##### Voice Recording

In order to perform the acoustic analysis, several voice recordings are carried out:

- “Platero y Yo“ text
- Held “a” letter. The patient must emit an “a” vowel with a moderate and clear voice tone. The acoustic analysis with the Jitter, Shimmer HNR and Pitch parameters is calculated on the basis of this recording.
- Low-pitched “a”. The patient must emit the lowest pitch which the patient can produce. This recording is used to calculate the frequency range of the patient.
- High-pitched “a”. The patient must emit the highest pitch which the patient can produce. This recording, together with the low-pitch data, is used to calculate the frequency range of the patient, measured in Hertz.
- Projected voice. The patient must emit a continuous sentence with strength and clarity.
- Sang voice. The patient must emit short words strongly.

At this point in the examination is not necessary to play the recording to process it, although it is recommended to listen to the recording quality before processing to ensure that it is correctly performed.

With the recordings carried out, the software is capable of calculating automatically the accoustic parameters of healthy patients, with functional pathologies, or even of laryngectomized patients.

#### Video/image analysis

**Figure 1:**
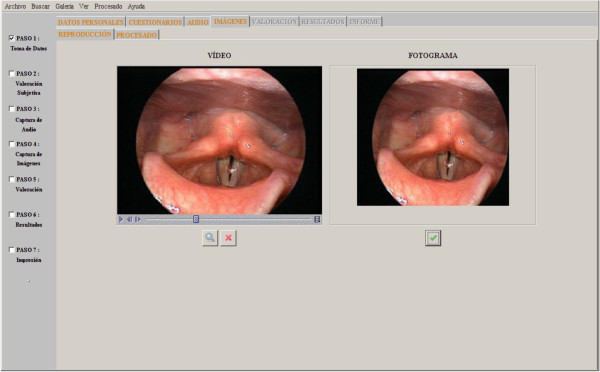
**Video analysis screen.**

In this section, the application allows to load, edit and process stroboscopic images, as it can be seen in Figure 1.

As mentioned above, the images to be analyzed by the application are VLG images. Stroboscopy can represent a vocal fold vibratory motion by means of shooting fixed individual images through a flash of light at different points of the cycles of the vocal cords vibrating at a frequency much lower than their real frequency of oscillation.

The stroboscopic image processing can provide us with objective information concerning the size of the glottal space at a certain point in time or the size of a polyp provided that this is the pathology the patient is suffering. It is important to bear in mind that given the features of the images all of this kind of measures have to be undertaken in pixels, not in centimeters since no real references are taken.

#### Assessment

**Figure 2:**
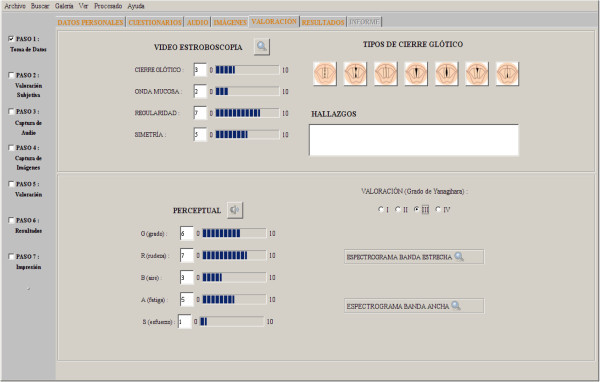
**Subjective VLG and Perceptual analysis.**

The final evaluation also requires a subjective (and integral) assessment by the doctor at a perceptual level and VLG analysis (see Figure 2).

##### VLG analysis

VLG Parameters taken into account in software application are described below:

- Glottal closure. Improper closure of the vocal folds, i.e. that the free edges of both vocal folds do not meet in the midline at its maximum adduction.
- Mucosal wave. The mucosal wave propagates from the subglottis and travels through the glottis to the upper surface of the vocal cord. It is a fine and delicate movement of the epithelium that spreads through the vocal cord. The normal mucosal wave is linear and continuously travels parallel to the edge of the vocal cord. Localized lesions can interfere with the mucosal wave propagation in the affected area.
- Regularity. The normal oscillation of the vocal cord should appear as periodical during the production of a sustained vowel. It is a severity classification parameter in the function. The regularity may be altered in spasmodic dysphonia, polyps and vocal cord cancer.
- Symmetry. It is the relation of vibration phase of a vocal cord related to the contralateral. The vocal cords are opened and closed symmetrically, i.e. vibrate in phase.

##### Perceptual analysis

There are several scales or profiles to parameterize a dysphonic voice, but the most commonly used is the GRBAS (Grade, Rouge, Astenic, Breathy and Strain) scale

- G-grade: measure of the overall severity of dysphonia.
- R-rough, hoarse: measure of the hoarseness, the dysphonia which is caused by the absence of mucosal wave vibration and irregularities due to mass effect on the vocal cord.
- Asthenic: measure of vocal fatigue, the inability to use the voice for long periods of time. The tone becomes lower pitched and the voice turns monotonous.
- B-Breathy: measure of the air in the voice. It is produced by the leakage of air between the vocal cords by an incomplete glottal closure. This type of voice typically appears in the vocal cord paralysis.
- S-Strain: measure of vocal effort, which corresponds to the hiperphonation or excessive tension of laryngeal muscles. It also produces difficulty in speaking, cervical muscles contraction, venous engorgement and mandibular projection. It is difficult to assess.

Each of the sections is rated on a scale of 0 to 3 points, where 0 would be normal, 1 mild, 2 moderate and 3 severe.

In general the parameters B and R, are easier to evaluate (audible), and typically indicate an organic pathology. A (anamnesis) and S (visual) are more difficult to evaluate, hence it is advisable to introduce only the GBR parameters.

##### Yanagihara Classification

The qualitative acoustic analysis is performed using a narrow band spectrogram of the sustained vowel /a/. Spectrograms are classified according to the degree proposed by Yanagihara ([Yanagihara 1967]).

- Grade I: Irregular harmonic components mixed with noise components, especially in the region of the formants of vowels.
- Grade II: Moderately hoarse voices with noise components in the second formants of vowels, predominant over the harmonic components, along with the appearance of some noise components in high frequency regions above 3000 Hz.
- Grade III: The second formants of vowels are completely replaced by components of noise, while the high frequency noise is intensified.
- Grade IV: There is loss of the periodic components of the first formants as a result of the present noise, and the high frequency noise is even more intensified.

## Results

**Figure 3:**
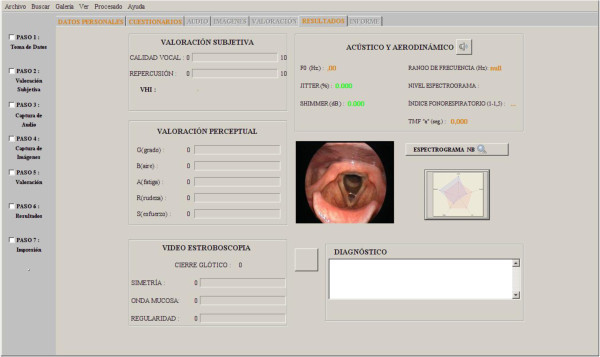
**Results Screen.**

This section shows the results obtained using the proposed tool with 25 patients during a year in otolaryngologist’s daily practice. Final results screen can be seen in Figure 3.

### Personal data analysis

Before assessing the results provided through/with the software, we review the grounds of the attendance to the consultation and the distribution by sex of the studied patients.

In this tests, 18 of the 25 patients included in the study were women (72%) and 7 men (28%) with an average age of 42 years and a range between 19 and 58 years (Standard Deviation 11.43).

In 18 cases the reason for consultation was exclusively dysphonia as the primary symptom; in 4 cases, vocal weariness and fatigue, and the remaining 3 were extravocal symptoms such as dry throat, neck pain, and feeling of a lump in the throat. The results for vocal use are described in Table 1.

**Table 1:** **Vocal use results**

Level I	Level II	Level III	Level IV
Exclusive or special use	Professional voice use	Non-vocal Professional	Workers without vocal use
4 cases	10 cases	8 cases	3 cases

### Subjective assessment analysis

The subjective assessment performed by the patient is a medical concept that is increasingly more important when making decisions and is of particular relevance for voice specialists. We have included 2 questionnaires in this software.

The first one (Table 2) is an abridged version with two basic questions scoring 0–10. These questions are: How do you think is your voice quality? (high quality = 10), and How do you think your voice affects your social life or work? (maximum effect = 10)).

**Table 2:** **Subjective assessment results**

Question 1	Good	Acceptable	Medium	Bad	Very Bad	
Subjective voice quality	-	7 cases	12 cases	4 cases	1 case	N=25
Question 2	**None**	**Little**	**Moderate**	**Serious**	**Severe**	
Socio-laboral subjective repercusion	4 cases	13 cases	5 cases	2 cases	1 cases	N=25

The second questionnaire is an expanded version consisting on the assessment of the Voice Handicap Index (VHI), which assesses through 30 items the physical, functional and emotional vocal pathology aspects (see Table 3), with a maximum score of 120 (0–40 to each group).

**Table 3:** **VHI results**

Classification of VHI in partial domains				
	Low (0–20)	Moderate (21–30)	Severe (31–40)	
Functional	4 cases	20 cases	1 cases	
Physical	8 cases	9 cases	8 cases	
Emotional	25 cases	8 cases	2 cases	
**VHI Classification in the overall assessment (sum of the partials)**				
	**Low (0–39)**	**Moderate (31–60)**	**Serious (61–90)**	**Severe (91–120)**
Global VHI	7 cases	12 cases	6 cases	-

### Morpho-functional study by laryngostroboscopy

The examiner assessed subjectively the glottal closure alterations, mucosal wave, regularity, and symmetry, with a score between 0 and 10, based on zero as the normal parameter value. In case of incomplete glottal closure, up to 5 different types of incomplete closures were evaluated, whose images appear with the rest of the stroboscopic parameters. The laryngoscopic images were captured in the Step 3 of the ANALYSIS VOX software: Image Capture.

Otolaryngologist diagnosis based on the patients’ videostroboscopy observation is the following:

- 52% of patients with nodules (13 cases)
- 12% of patients with vocal polyp (3 cases)
- 16% of patients with Reinke’s edema (4 cases)
- 12% of patients with Sulcus vocalis (3 cases)
- 8% of patients without fuctional injury (2 cases)

Reviewing Table 4, the glottal closure was more impaired in case of polyps and nodules (8 and 7 respectively) as they are free edge exophytic lesions. The mucosal wave was altered in all cases except in patients with Reinke's edema. The unilateral polyp and sulcus vocalis are the injuries which obtained higher scores. The sulcus vocalis and vocal polyp lesions shown the highest impairment of the regularity of the mucosal wave (high score obtained).

**Table 4:** **Morpho-functional study results**

Rating 0-10	Glottal closure	Mucosal wave	Regularity	Symmetry
Nodules	7	3	3	2
Vocal polyp	8	7	7	9
Reinke's edema	1	0	2	1
Sulcus vocalis	8	7	8	9
No injury (functional)	5	3	6	6

The nodules and Reinke's edema are the injuries that have less impact on the symmetry parameter (Eysholdt et al. [2003]).

Four of the 25 cases show complete closure (no glottal hiatus). The most frequent type of hiatus was the hourglass glottal hiatus (13 cases), followed by the posterior hiatus (4 cases) and the irregular (4 cases).

**Figure 4:**
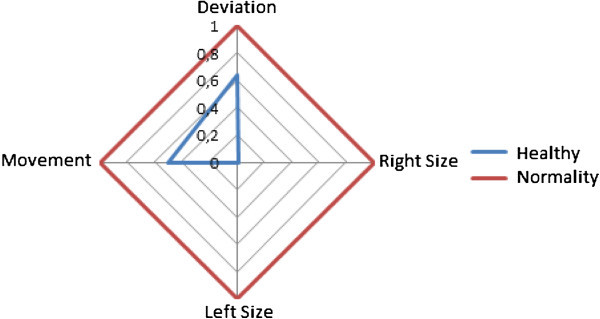
**VLG objective parameters graphic results.**

The software also provides a graphic representation of the parameters calculated automatically (as it can be seen in Figure 4).

- Right Size and Left Size.These parameters make reference to the size in pixels the pathology has, in the event of being one.
- Deviation. This parameter meassures the angle formed with the right and left vocal fold.
- Movement. Combines the measures carried out to parameterize the movement of the vocal folds (Méndez et al. Mendez et al. [2012]).

#### Perceptual analysis of dysphonia

The results of the evaluation of the perceptual analysis of speech are shown in Table 5:

**Table 5:** **Perceptual analysis results**

Patients rated according to the perceptual analysis of dysphonia					
Rating from 0 to 10, from low to severe grade					
	0-1-2 points	3-4 points	5-6 points	7-8 points	9-10 points
**G. Grade**	6 cases	12 cases	4 cases	3 cases	-
**R. Rouge**	5 “	14 “	3 “	3 “	-
**A. Asthenic**	3 “	11 “	5 “	3 “	3 cases
**B. Breathy**	4 “	10 “	6 “	4 “	1 “
**S. Strain**	2 “	8 “	7 “	7 “	1 “

### Acoustic and spectrographic analysis

The vocal acoustic analysis is performed by emitting the letter /a/ in a comfortable way in tone and intensity, with the microphone at a distance of 15 cm and an angle of 45° in relation to the mouth.

We chose a homogeneous fragment of the recording (approximately 50 vibratory cycles) corresponding to a second of the middle portion of each vocal register and we proceeded to its analysis with the program included in our software tool.

Once the signal was converted from analogic to digital, the program calculated the following parameters: Fo, Jitter, Shimmer and Glottal Noise (HNR).

The values of the 25 patients are shown in Table 6. They express the values for the mean, standard deviation, confidence intervals at 95% as well as the maximum and minimum values of each of the variables.

**Table 6:** **Acoustic analysis results**

Acoustic parameter values of the 25 patients				
Variables	Mean	Standard Deviation	Confidence Intervals (95%)	Max/Min Values
Fo mean	178,12	31,33	165,45 a 204,63	285,41/110,51
Fo Male	127,56	24.12		
Fo Female	232,87	29,74		
Jitter	0,35	0,95	0,20 a 0,29	0,45/0,12
Shimmer	4,10	2,51	3,42 a 5,25	10,10/1,30
HNR	23,12	3,30	17,80 a 23,95	27,58/12,63

Regarding the results for spectrogram classification of the 25 records, Yanagihara criteria for classification, based on the absence of harmonics in the spectrum, signal void in the spectrogram without noise substitution and other pathological paths were considered.

The results were classified as follows: type I: 3 cases, type II: 6 cases, type III: 7 cases and type IV: 4 cases. Finally, three records were classified as normal.

### Analysis of aerodynamic efficiency

The results of the measurement of aerodynamic efficiency shown that the mean of the maximum phonation time results (MFT) for the vowel /a/ was 11.42 seconds, with a range between 6 and 28 (standard deviation: 5.60). Values below 10 seconds should be considered pathological. Pathological cases are caused by 2 circumstances: either low volume bronchial pathology or by laryngeal pathology with glottic efficiency loss, as in the case of our patients.

Phonorespiratory Index: is calculated by MFT "s" / MFT"a". The normal limit is between 1.4 and 1.5. Values greater than 1.5 are related to defects of closure due to glottic incompetence. In 20 of the 25 cases, the phonorespiratory index was pathological (greater than 1.5), with a mean of 1.7 and a range between 1.4 and 1.9 (standard deviation 4.40).

### Vocal profile

**Figure 5:**
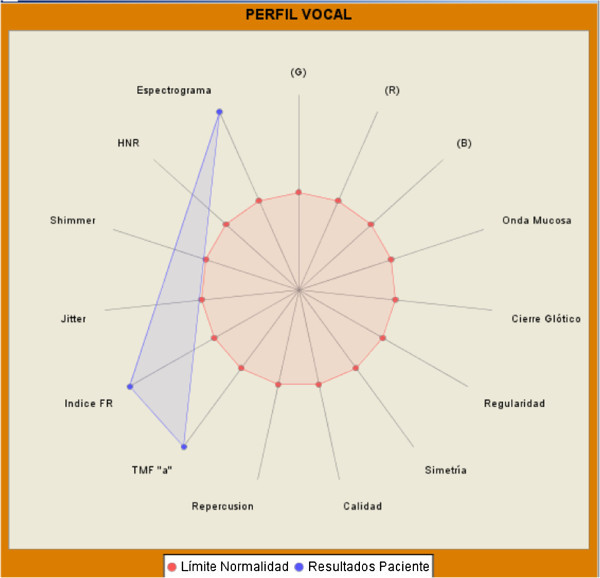
**Example of vocal profile of a pathological patient.**

The vocal profile is a visual summary of the data analyzed by this software (see Figure 5).

It is a multidimensional diagram which represents the layout of the most important parameters of the analysis. Those who are within the normal threshold, appear within the demarcation of the red circle, while the pathological values appear located outside the threshold circle.

Thus a multidimensional graph of the patient is obtained in which the larger the area of the graph, the greater the vocal lesion, and the smaller the area of the diagram indicates a lower vocal pathology.

The parameters included in the vocal profile are:

1. Subjective quality
2. Subjective repercussion
3. Perception G
4. Perception R
5. Perception B
6. MFT
7. Jitter
8. Shimmer
9. HRN
10. Stroboscopy: Close
11. Stroboscopy: Mucosal wave
12. Stroboscopy: Regularity
13. Stroboscopy: Symmetry

### Audiovisual gallery

**Figure 6:**
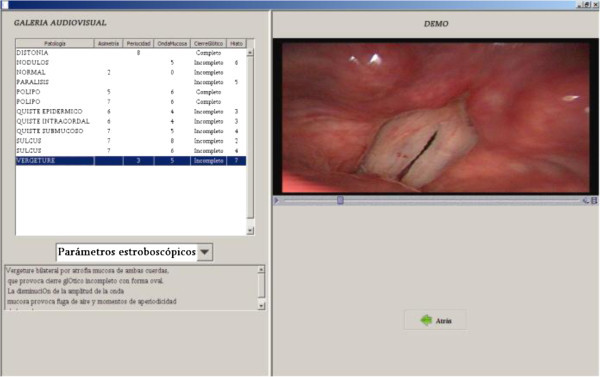
**Audiovisual Gallery Screen.**

The audiovisual gallery is a virtual library of stroboscopic images , recorded voices and their associated diagnostic with primarily didactic purposes, since performing these exercises helps us to properly assess the patient data that will be studied in the program (see Figure 6).

It is advisable to consult the audiovisual gallery to obtain the maximum efficiency from the studies. It consists of four libraries:

1. Stroboscopic Parameters: Models of stroboscopic images are shown with the scores of the various parameters.
2. Vocal Pathology: consist of laryngoscopic video recordings with their corresponding lesion diagnoses.
3. Perceptual Evaluation: includes audio recordings of pathological voices with evaluations of the GRBAS parameters.
4. Spectrograms: standard images consisting of different types of pathological spectrograms (I-IV) according to the criteria of Yanagihara.

### Subjective results

Beside the objective results corresponding to the acoustic parameters analyzed, or related to the features of the vocal cords, the software has been reviewed and analyzed by experts in otolaryngology which this way help the authors in the continuous improvement of the application.

The feedback of the otolaryngologists was collected through some opinion questionnaires attached below (Table 7), and from which some conclusions were extracted.

**Table 7:** **Survey made to otolaryngologists**

Item	Average Marks
The use of the AnalisisVOX software is easy	3,53
Friendly interface	3,14
The acoustic parameters analyzed are appropriate	3,97
The graphic representation of the acoustic parameters is appropriate	4,18
The analyzed parameters of the vocal fold images are appropriate	3,82
The graphic representation of the parameters obtained automatically through the images is appropriate	4,5
Quality of the generated reports	4,47

Each one of the evaluated Items has been valued from 1 to 5, being 5 totally agree and 1 totally agree, and 1 totally.

As it can be seen in the Table, otolaryngologists evaluate especially well the graphic representations provided by the software (4,55 and 4,18), which allow to detect abnormalities in the voice and/ or in the vocal cords in a very visual way.

Regarding the ease of use of ANALISISVOX and its interface the evaluation remains good 3,53 and 3,14 respectively. The adaptation of the software to the management of the software is below one week, and the provided feedback is more related to the inclusion of new functionalities which, with problems related to those developed ones.

## Conclusions

There are plenty of programs ([Matassini and Manfredi 2002]; [Hadjitodorov and Mitev 2002]; Gelzinis et al. [2008]) which serve as a tool for doctors who study the voice during an appointment. In most cases these are applications that allow you to record your voice and emit certain frequency parameters, or to analyze independently the features of the voice and of the vocal fold images. However, these applications are insufficient (as it can be seen in a comparison in Table 8) and often inefficient even when performing a medical consultation, since they do not provide complete information but only part of it. Therefore, doctors are forced to perform the exploration separately, gathering the information with different methods, generating files and documents from different software programs that fill the useful computer memory and in addition slowing the study process.

**Table 8:** **Comparative features of ANALISISVOX**

Features of the current applications	Features of the ANALISISVOX application
**Insufficient.** They do not provide the complete information the specialist doctor needs when carrying out a medical consultation.	**Complete.** It is integrated within a single application, just the needed analyses the specialist doctor requires to diagnose (according to the ELS protocol).
**Inefifcient.** They do not provide the specific information necessary for the specialist doctor.	**Efficient.** An automated system is established, sequential, which makes the consultation easy and rapid /quick.
Lead to the specialist doctor to carry out the exploration in parts, gathering the information through different methods, generating files and documents of different programs which occupy usable memory of the computer, and moreover slowing down the process of the study.	It can be accessed to the files of any patient, without having to seek manually on the hard disk any kind of information
Too technical when showing the calculated information.	Shows the values obtained in a graphic and easy way to digest.

It is noticeable that there is great interest aroused by these applications in the field of ENT, speech pathologists and speech therapists, but the big question is how many of them respond to the needs of specialists. In the case of the proposed tool the vision of the experts who have participated throughout the development process has been implemented, therefore its applicability is granted.

The proposed application performs a post-processing of the signals supplied during a clinic session, and it unifies concepts and results which are usually analyzed independently. The preparation of all reports with all the provided objective parameters allows the study of the evolution of the patients during the rehabilitation process or after surgery. We could even evaluate the effectiveness of treatments and suggest modifications.

## References

[CR1_98] Braunschweig T Flaschka J Schelhorn-Neise P Döllinger M High-speed video analysis of the phonation onset, with an application to the diagnosis of functional dysphonias Med Eng Phys 2008 30 1 59 66 10.1016/j.medengphy.2006.12.007 17317268

[CR2_98] Eysholdt U Rosanowski F Hoppe U Vocal fold vibration irregularities caused by different types of laryngeal asymmetry European Arch Otorhinolaryngol 2003 260 1 412 417 10.1007/s00405-003-0606-y 12690514

[CR3_98] García B Ruiz I Méndez A Mendezona M Objective characterization of oesophageal voice supporting medical diagnosis, rehabilitation and monitoring Comput Biol Med 2009 39 97 105 10.1016/j.compbiomed.2008.11.009 19159870

[CR4_98] Gelzinis A Verikas A Bacauskiene M Automated speech analysis applied to laryngeal disease categorization Comput Methods Programs Biomed 2008 91 1 36 47 10.1016/j.cmpb.2008.01.008 18346812

[CR5_98] Hadjitodorov S Mitev P A computer system for acoustic analysis of pathological voices and laryngeal diseases screening Med Eng Phys 2002 24 6 419 429 10.1016/S1350-4533(02)00031-0 12135650

[CR6_98] Hsiung M-W Pai L Wang H-W Correlation between voice handicap index and voice laboratory measurements in dysphonic patients Eur Arch Otorhinolaryngol 2002 259 2 97 99 10.1007/s004050100405 11954941

[CR7_98] Kim DY Kim LS Kim KH Sung MW Roh JL Kwon TK Lee SJ Choi SH Wang SG Sung MY Videostrobokymographic analysis of benign vocal fold lesions Acta Otolaryngol 2003 123 9 1102 1109 10.1080/00016480310001880 14710916

[CR8_98] Kiritani S Honda K Imagawa H Hirose H Simultaneous high-speed digital recording of vocal fold vibration and speech signal Proc IEEE ICASSP'86 1986 11 1633 1636

[CR9_98] Kiritani S Hirose H Imagawa H High-speed digital image analysis of vocal cord vibration in diplophonia J Speech Commun 1993 13 23 32 10.1016/0167-6393(93)90056-Q

[CR10_98] Lee JS Kim E Sung MW Kim KH Park KS A method for assessing the regional vibratory pattern of vocal folds by analysing the video recording of stroboscopy Med Biol Eng Comput 2001 39 3 273 278 10.1007/BF02345279 11465879

[CR11_98] Matassini L Manfredi C Software corrections of vocal disorders Comput Methods Programs Biomed 2002 68 2 135 145 10.1016/S0169-2607(01)00161-4 11932030

[CR12_98] Mendez A Ismaili Alaoui EM García B Ibn-ElHaj E Glottal Space Segmentation from Motion Estimation and Gabor Filtering 2009 USA Minneapolis 10.1109/IEMBS.2009.5332612 19963652

[CR13_98] Mendez A, Lopetegui E, Garcia B: *Vocal Folds Paralysis Classification using FLDA and PCA algorithms suported by an Adapted Block Matching Algorithm*. Rome, Italy; 2012. [*Proceedings of ISCCSP12*]

[CR14_98] Nerrière E Vercambre ML Gilbert F Kovess-Masféty V Voice disorders and mental health in teachers: a cross-sectional nationwide study BMC Publ Health 2009 9 370 10.1186/1471-2458-9-370 PMC2762990 19799781

[CR15_98] Núñez BF Validación de la versión traducida al español del índice de incapacidad vocal (voice handicap index) Acta Otorrinolaringol Esp 2007 58 9 385 10.1016/S0001-6519(07)74953-1 17999901

[CR16_98] Rodríguez-Parra MJ Adrián JA Casado JC Voice therapy used to test a basic protocol for multidimensional assessment of dysphonia J Voice 2009 23 3 304 318 10.1016/j.jvoice.2007.05.001 17658721

[CR17_98] SEORL 2012. http://www.seorl.net/ Accessed 26 August

[CR18_98] Weigelt S Krischke S Klotz M Hoppe U Köllner V Eysholdt U Rosanowski F Voice handicap in patients with organic and functional dysphonia HNO 2004 52 8 751 756 10.1007/s00106-003-1039-z 15042304

[CR19_98] Yanagihara N Significance of harmonic changes and noise components in hoarseness J Speech Hear Res 1967 10 531 541 6081935 10.1044/jshr.1003.531

